# Quantifying physiological stability in the general ward using continuous vital signs monitoring: the circadian kernel density estimator

**DOI:** 10.1007/s10877-023-01032-2

**Published:** 2023-06-02

**Authors:** Søren S. Rasmussen, Katja K. Grønbæk, Jesper Mølgaard, Camilla Haahr-Raunkjær, Christian S. Meyhoff, Eske K. Aasvang, Helge B. D. Sørensen

**Affiliations:** 1 Biomedical Signal Processing & AI Research Group, Digital Health Section, Department of Health Technology, Technical University of Denmark, Ørsteds Plads, Building 345B, 2800 Kgs, Lyngby, Denmark; 2 Department of Anaesthesia and Intensive Care, Copenhagen University Hospital - Bispebjerg and Frederiksberg Hospital, Copenhagen, Denmark; 3 Department of Anaesthesiology, the Center for Cancer and Organ Diseases, Copenhagen University Hospital - Rigshospitalet, Copenhagen, Denmark; 4 Department of Clinical Medicine, University of Copenhagen, Copenhagen, Denmark

**Keywords:** Kernel Density Estimation, Continuous vital signs monitoring, Stability index, Physiological deterioration, General ward

## Abstract

Technological advances seen in recent years have introduced the possibility of changing the way hospitalized patients are monitored by abolishing the traditional track-and-trigger systems and implementing continuous monitoring using wearable biosensors. However, this new monitoring paradigm raise demand for novel ways of analyzing the data streams in real time. The aim of this study was to design a stability index using kernel density estimation (KDE) fitted to observations of physiological stability incorporating the patients’ circadian rhythm. Continuous vital sign data was obtained from two observational studies with 491 postoperative patients and 200 patients with acute exacerbation of chronic obstructive pulmonary disease. We defined physiological stability as the last 24 h prior to discharge. We evaluated the model against periods of eight hours prior to events defined either as severe adverse events (SAE) or as a total score in the early warning score (EWS) protocol of ≥ 6,  ≥ 8, or ≥ 10. The results found good discriminative properties between stable physiology and EWS-events (area under the receiver operating characteristics curve (AUROC): 0.772–0.993), but lower for the SAEs (AUROC: 0.594–0.611). The time of early warning for the EWS events were 2.8–5.5 h and 2.5 h for the SAEs. The results showed that for severe deviations in the vital signs, the circadian KDE model can alert multiple hours prior to deviations being noticed by the staff. Furthermore, the model shows good generalizability to another cohort and could be a simple way of continuously assessing patient deterioration in the general ward.

## Introduction

It is known that hospital deaths and severe complications in many cases are both predictable and preventable [1–3]. Track-and-trigger (T&T) systems, such as the early warning scoring (EWS) protocol, are therefore a well-established part of the standard care practice in the general wards at hospitals in Europe and the United States of America. The EWS protocol consists of tracking physiological parameters (respiration rate (RR), peripheral oxygen saturation (SpO2), blood pressure, heart rate (HR), etc.) and triggers a specific intervention based on the magnitude of the physiological derangement. It relies on point measurements of physiological parameters conducted at intervals of up to 12 h [4, 5]. Even though, the EWS protocol was implemented in order to obtain an objective and ‘continuous’ system for early detection of patient deterioration, documentation of its impact on mortality and morbidity is lacking due to factors such as missing records, digit preferences, and high intervals between measurements [5–7].

The technological advances seen in recent years has the potential for changing the monitoring paradigm at the general ward. Whereas continuous monitoring was previously restricted to specialized sections such as the intensive care unit (ICU) and cardiology wards, wearable technologies and increased computational power now allow for monitoring vital signs continuously to be done in the general wards, while patients can still adhere to the enhanced recovery after surgery protocols of early mobilization [8, 9]. The implementation of wireless continuous monitoring systems in general wards could potentially solve some of the aforementioned issues linked to EWS protocols, but it is still awaiting implementation on a wide scale [8]. Moreover, continuous monitoring paves the way for high resolution data to allow for computer aided decision-making and support and potentially a novel understanding of vital signs and outcomes including evidence-based alerts.

With more continuous clinical vital signs data available, the amount of research of analysis of this has increased in recent years. As larger samples of data become available, the use of big data approaches for analyzing health care data will aid in discovering trends and patterns, that will certainly change the way monitoring in hospitals are done today [10]. In continuous monitoring of patients in general wards, simple alerts about every vital sign deviation cannot be implemented because of limited staff availability and the plethora of alerts that will result in alarm fatigue. Labeling of relevant deviations that are related to important adverse outcomes are therefore needed. There is yet no ‘gold standard’ for the labeling of continuous vital signs data which creates issues with applying classic machine learning. It is not feasible to retrospectively label the vital signs data itself by visual inspection, as is the standard practice in analysis of most other types of biomedical signals (e.g., Electrocardiography, imaging and Electroencephalography). Instead, others have suggested labeling the data by retrospective analysis of manual observations and case-notes [11, 12], identification of adverse events during the hospitalization [13–15], admission to the ICU [16, 17], along with a comparison to T&T systems [18, 19]. Thus, creating the labels based on information other than the signals themselves.

The analysis of vital signs data is heterogenous. Some studies used heuristically set thresholds either fixed [14–16] or adaptive [17] to detect events and compare these to adverse events. Other studies suggest splitting the data into a positive and negative class based on the time to an event and addressing it as a two-class problem [20, 21]. Finally, other studies address the problem using novelty detection, either by the use of gaussian processes [11, 22], state-space models [23], or distribution models [11, 24].

Given the above, we aimed to investigate detection of physiological deterioration by novelty detection. Kernel Density Estimation (KDE) allows for this by constructing a density of normal observations and evaluating new observations in relation to this. Though the use of KDE on vital signs data is not novel, none of the analyzed studies seemed to incorporate the natural fluctuations in the vital signs given by the circadian rhythm into the models [11, 24]. The circadian rhythm greatly effects several vital signs parameters and have shown to be disturbed in patients with severe physiological deterioration [25, 26].

We hypothesized that incorporation of this in the model would lead to a better discrimination margin between normal and abnormal physiology. We hypothesize that implementing a circadian KDE model based on continuous monitoring of vital signs and computing a stability index using novelty detection can aid in identifying physiological deterioration earlier than the current practice.

## Methods and procedures

This study was done as part of the Wireless Assessment of Respiratory and circulatory Distress (WARD) project. The data from two different observational studies with continuous monitoring of vital signs were used in this study. Both studies were registered at http://ClinicalTrials.gov (NCT03491137, NCT03660501) after written approval from the Regional Ethics Committee (H-17033535, *H-18026653*). All patients had given informed consent prior to inclusion [15, 19].The primary analysis was done using data from a cohort of 491 postoperative patients in the period following major abdominal cancer surgery [15]. The secondary analysis was done using data from a cohort of 200 patients admitted with acute exacerbation of chronic obstructive pulmonary disease (AECOPD) [19]. The secondary analysis was included to test the generalizability of the model, as the two cohorts are believed to be substantially different due to the different reasons for admission and thus different physiological representations of possible deterioration. All patients were planned monitored for up to 96 h or until discharge.

### Data collection

Continuous monitoring used the Isansys Patient Status Engine, an end-to-end platform for wireless monitoring of patients. The system consisted of the sensors: (A) Isansys Lifetouch (Isansys Lifecare, Oxfordshire, UK)—a single-lead ECG device that records HR (1/60 Hz), RR (1/60 Hz), from electrocardiogram (B) Nonin WristOx 3150 (Nonin Medical Inc., Minnesota, USA)—a finger mounted pulse oximeter that records SpO2 (1/60 Hz) (C) Meditech BlueBP-05 (Meditech Ltd., Hungary)—a blood pressure cuff measuring systolic and diastolic blood pressure (Sys. BP and Dia. BP) every 30 min during the daytime (07:00 to 22:00) and every hour during the nighttime (22:00 to 07:00). The recorded data were transmitted via Bluetooth to a bed side gateway from which the data were sent via Wi-Fi to a local server database at the hospital.

### Preprocessing and computing features

The continuous variables recorded in the study, is not suited for a KDE in the raw format, as there is a high occurrence of smaller periods of missing data and as the devices are prone to artefacts. Therefore, we decided to create observations in a window-based way, such that features were computed over an interval of data points. Each observation would then represent a sample in the KDE model. The flow of computing features is shown in Fig. 2.

We selected the four ‘standard’ modalities in vital signs monitoring, Sys. BP, HR, RR, and SpO2. Apart from the systolic BP, the remaining modalities are continuous signals (1/60 Hz) and prone to artefacts from sensors displacement and movement. We have found this to be a common source of errors, and applied a moving median filter with a size of 4 min as proposed by Sow et al. [27].

After filtering, we split the signals into observations in a window-based approach. Three window lengths were used with the length of 30, 60, and 120 min, and the windows were moved 10 min for each observation thus giving an overlap in the observations. Each observation was used as a sample for the model, making it possible to compute a stability index every 10 min independent of the window length. Within each window, the mean value of each of the four signals was computed as the only feature. This resulted in each observation consisting of four features. We labeled the observations to either belong to the physiologically stable class, or one of four event classes. The timestamp of the observations was set to be at the end of the window, such that the time to an event would reflect the moment the observation could be computed in a real-life setting. Observations not belonging to any of the classes, was discarded.

### Definition of the physiologically stable class

**Fig. 1:**
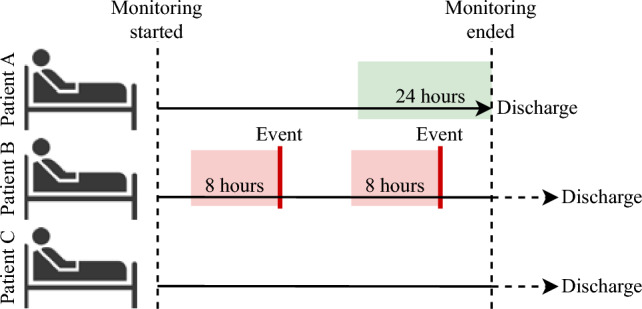
Visualization of labeling. Patient A represents a patient with no SAEs during the monitoring period and with monitoring ending due to discharge. The green area shows the last 24 h of monitoring which is defined to be physiologically stable. Patient B represents a patient with two events occurring during monitoring. For each event, 8 h prior to the event is defined as physiologically unstable (red area). Patient C represents a patient with no events but with the time of discharge occurring after monitoring has ended. This patient is removed from the analyses

In defining the stable physiology, we aimed to select the period of monitoring that would represent the patients which followed the optimal trajectory of recovery at their best physiological state. We defined this as the last 24 h of monitoring for patients for whom the monitoring ended due to discharge from the hospital if and only if they had not had any Severe Adverse Events (SAE) during their stay. All valid observations within this 24-h period would be labeled as stable. This period was chosen as the patients fulfilling these criteria would have a short hospitalization (as monitoring ended after 96 h), and we assumed that the last 24 h of their monitoring would represent the time of their stay where they were under the least amount of surgical stress. The definition of physiological stability is visualized in Fig. 1. in patient A, where the green area represents the region defined as physiologically stable. If a patient did not have any SAEs but was discharged after monitoring ended (as show in patient C in Fig. 1) data from this patient was excluded for the further analysis, as it would not fit the criteria for any class. This definition resulted in data from 161 (33%) patients from the postoperative cohort and 81 (41%) in the AECOPD cohort being physiologically stable, as shown in Table 1.

**Table 1:** Number of patients in each of the defined classes for both the surgical and AECOPD cohort

Class	Surgical cohort (n = 491)	AECOPD cohort (n = 200)
Physiologically stable	161 (33%)	81 (41%)
SAE	71 (14%) 102 events	54 (27%) 73 events
EWS6 event	99 (20%) 313 events	28 (14) 95 events
EWS8 event	21 (4%) 62 events	3 (2%) 7 events
EWS10 event	4 (1%)12 events	None

The number in parentheses denotes the percentage of the full cohort. The total number of events within each event class is listed below. Some patients might have more than one event

We chose to use data which we assumed would represent stable physiology as the normal class, as (1) it is believed to have a homogeneous expression of the vital signs in relation to abnormalities, which differ dependent of the mechanism behind (respiratory, cardiovascular, infectious, etc.) (2) it is overrepresented within the patients monitored in relation to abnormalities, and (3) it would allow for us to create a metric of stability centered around physiological stability.

### Definition of the physiological unstable classes

In order to evaluate the model against physiological deterioration, we defined four unstable events from the patients’ electronic health records, of which three were defined from the manually reported EWS measurements and one was based on the reported SAEs during a patient’s stay. For each event type, the eight hours of data leading up to the event was considered physiologically unstable, and observations within this period were labeled as such. This is shown in Fig. 1 in patient B, where data from the red areas are labeled as physiologically unstable.

SAEs during the monitoring period were registered retrospectively from the electronic health records as part of the studies [15, 19]. SAEs were defined as any untoward medical occurrences that were either life-threatening, requiring prolonged hospitalization or resulting in persistent or significant disability in accordance to the International Conference on Harmonization—Good Clinical Practice (ICH-GCP) guidelines [28]. The timestamp of the SAE was set to the time that evidence was present, e.g. if an SAE was confirmed by a blood test, the timestamp was set to the time the blood sample was taken and not when the diagnostic result was available. The number of SAEs in each cohort is listed in Table 1.

From the EWS, three events were created based on the total sum of the scores given by the values of the measured vital signs. The measurements used for the events were obtained in the standard care according to local regulations at the hospitals [29]. We chose three events defined as a total EWS score of ≥ 6,  ≥ 8, or ≥ 10. This represented scores which would require the nurse notifying a medical doctor at different levels urgency and magnitude of deterioration. The timestamp was set to the time noted by the staff conducting the measurements. Several of the measurements had one or more missing variables which were set to a score of zero. The number of events within each score are shown in Table 1.

### Multivariate kernel density estimation

The KDE is a non-parametric method for estimating a probability density function of a random variable using kernel smoothing. The kernel density of a set of random samples is created by applying a kernel function to each random sample and summing these to create the density. Thus, regions with large number of observations will get result in a high density estimate and regions with a sparse number of observations a low estimate. Hence, we can create the KDE from a set of observations representing physiological stability and achieve a density where the evaluation any given point will reflect the likelihood of that point to come from observations of stable physiology.

We defined our training set of $n$ observations of stable physiology, $\mathbf{X}=\left[{\mathbf{x}}_{1},\dots ,{\mathbf{x}}_{n}\right]$, where each observation, ${\mathbf{x}}_{i}$, was a vector of the four features computed, ${\mathbf{x}}_{i}={\left({\overline{Sys. BP}}_{i}, {\overline{HR} }_{i},{\overline{SpO2} }_{i}, {\overline{RR} }_{i}\right)}^{\mathrm{\top }}$. For any new observation, $\mathbf{x}\mathbf{^{\prime}}={\left(\overline{Sys. BP}, \overline{HR },\overline{SpO2 }, \overline{RR }\right)}^{\mathrm{\top }}$, we quantified the resemblance with normal physiology by the gaussian multivariate kernel density estimate given by [30]

1 $$\hat{f}\left( {{{\varvec x}^{\prime}}|{\varvec{X}},{\varvec{H}}} \right)\, = \,\frac{1}{n}\mathop \sum \limits_{i = 1}^{n} K_{{\varvec{H}}} \left( {{{\varvec x}^{\prime}} - {\varvec{x}}_{i} } \right)\, = \,\frac{1}{{n\left( {2\pi } \right)^{d/2} \left| {\varvec{H}} \right|^{1/2} }}\mathop \sum \limits_{i = 1}^{n} e^{{ - \frac{1}{2}\left( {{{\varvec x}^{\prime}} - {\varvec{x}}_{i} } \right)^{ \top } {\varvec{H}}^{ - 1} \left( {{{\varvec x}^{\prime}} - {\varvec{x}}_{i} } \right)}},$$

where the dimension, $d=4$, and $\mathbf{H}$ is the $d\times d$ bandwidth matrix, a nonsingular matrix. [31]. We defined the abnormality of any new observation, ${\mathbf{x}}^{^{\prime}}$, by the stability index, $\mathrm{SI}\left({\mathbf{x}}^{^{\prime}}\right)$, given by

2 $${\text{SI}}\left( {{\mathbf{x^{\prime}}}} \right)\, = \, - \,\log \left( {\hat{f}\left( {{\mathbf{x^{\prime}}}{|}{\mathbf{x}},{\mathbf{H}}} \right)} \right).$$

Hence, the stability index was used to represent how likely any given observation was to be physiologically stable where a stability index close to zero indicates an observation of stable physiology and increasing stability index indicates an observation of increasing deviation in the physiology. By comparing the stability index to a threshold, $\lambda$, a simple classification can be constructed to identify the deteriorating patient as

3 $$y^{\prime}\, = \,\left\{ {\begin{array}{*{20}c} 1 & {{\text{if}} \;SI\left( {{\mathbf{x^{\prime}}}} \right) > \lambda } \\ 0 & {{\text{otherwise}} } \\ \end{array} } \right. .$$

The only free parameter in the gaussian KDE is the bandwidth matrix, $\mathbf{H}$. The bandwidth matrix controls the smoothness of the kernels, with the smoothness of the estimate getting larger as bandwidth increases. Many different methods exist for selecting the bandwidth including using Silverman’s rule [32], Scott’s rule [31], or optimizing the parameters in a cross-validation setup [30]. In the current study, the bandwidth matrix, $\mathbf{H}$, was set to be proportional to ${{\varvec{\Sigma}}}^{1/2}$ in accordance with Scott’s Rule, as suggested by Härdle et al. [30], such that

4 $${\hat{\mathbf{H}}}\, = \,n^{{1/\left( {d + 4} \right){ }}} {\hat{\mathbf{\Sigma }}}^{1/2}.$$

### The proposed circadian KDE model

**Fig. 2:**
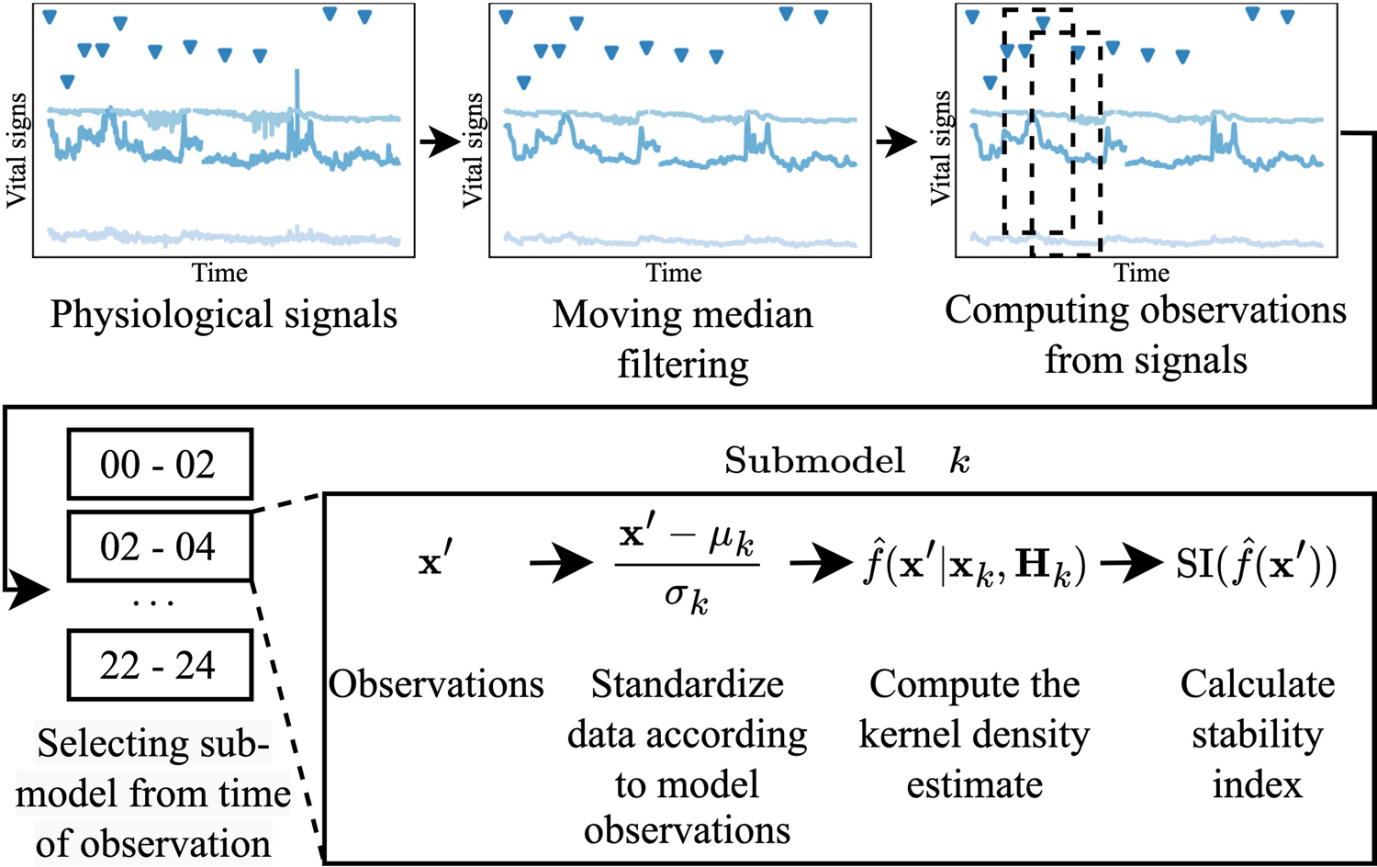
Flow diagram of the proposed circadian KDE model. The plots in the top row illustrate the continuously measured vital signs in before and after filtering as well as applying the windows for computing the observations. The bottom row shows the flow in the novel circadian KDE model. ${\upmu }_{\mathrm{k}}$ and ${\upsigma }_{\mathrm{k}}$ are the standardization parameters, ${\mathbf{x}}_{\mathrm{k}}$ are the model observations, and ${\mathbf{H}}_{\mathrm{k}}$ is the bandwidth matrix, all specific for the submodel, $\mathrm{k}$. *SI* stability index

To incorporate the oscillations of normal vital signs values during the day and night into the models, we proposed a separate model setup based on the time of day, such that multiple KDE models were fitted using only observations from a given time interval during the day. We heuristically chose 12 intervals (spanning 00-02, 02-04, …, 22-24), as this was believed to provide enough intervals to capture the circadian oscillations, but at the same time ensure that there would be a sufficient number of observations within each. Within each model, observations were standardized to zero mean unit variance based on training observations, and the bandwidth matrix were computed. We chose the separate model setup, as there is no ordinal scale in the time of day. The flow of the circadian configuration is shown in Fig. 2

By creating the model in a circadian setup, we aimed to achieve two things (1) that the natural circadian oscillations in vital signs would not lead to the observations appearing abnormal during the extreme periods of the day, e.g. normally low heart rate during the night appearing abnormal in a model fitted with day-time observations, and (2) that the distribution of the stable physiology would be as narrow as possible to avoid deviations being ‘hidden’ in the circadian oscillations, e.g. a high heart rate during the night being ‘hidden’ by a model fitted with day-time observations.

### Experimental setup

The experimental setup was intended to evaluate the different configurations of the model to compare these. We evaluated the circadian KDE with the three different window sizes (30 min, 60 min, 120 min).

**Fig. 3:**
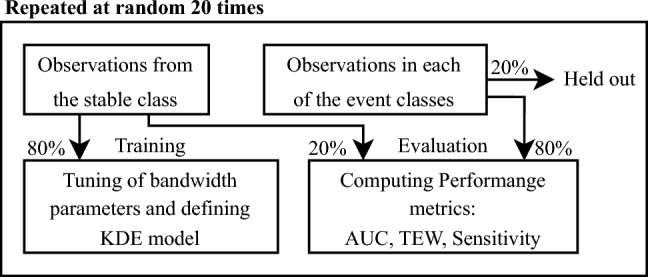
Setup of each iteration in the experimental setup showing the splits of observations for training and evaluating the models. Observations are chosen at random but with the constraint that observations from one patient cannot be present in both training and evaluation. The held-out samples are not used in the iteration

In order to estimate the performance of a model configuration, we divided the dataset of the surgical patients into training and test sets, with the training set containing 80% of the observations from the stable class and the test set containing the remaining 20% along with 80% of the observations from each of the event classes. The remaining 20% of the event observations were held out. This analysis was repeated 20 times in a setup shown in Fig. 3, to estimate the uncertainty in the results. Each iteration would therefore consist of splitting, retraining, and reevaluating the performance of the model, each time with different training and test sets. All splits were done at random and such that no observations from the same patient would be present in both training and test set. This is important, as the training and test set would not otherwise be truly independent.

For patients in the AECOPD cohort, we evaluated the model in the same paradigm, with slight modifications. For each repetition, the models were fitted using 80% of the stable observations from the postoperative cohort. The test was done on 80% of both the stable observations and the observations from each event class from the AECOPD cohort. This was likewise repeated 20 times.

For each repetition we constructed the receiver operating characteristics (ROC) curve based on the classification results and computed the area under the ROC (AUROC). In addition we computed the Time of Early Warning (TEW), adopted from Colopy et al. [12] with modifications. The TEW is computed for each event as the time to the event at the earliest positive classification or zero if no positive classification is done. The reported TEW is the average across all occurrences within each event class. We also visualize the TEW in a curve with the TEW against the false positive rate (FPR) to demonstrate the early possible intervention in relation to the number of false alerts. In order to use the TEW as a single metric, we used the value of the TEW at FPR = 0.1. Furthermore, we computed the sensitivity at FPR = 0.1, such that the accuracy of the model would be comparable with the TEW.

## Results

### Available data for modelling

The number of valid observations within each class, i.e. with all features present, is shown in Table 2. As can be seen, the number of complete observations within the stable class is 7,980, 11,183, and 11,994 for a window size of 30, 60 and 120 min respectively, which corresponds to 32%, 45%, and 51% of the total number of observations computed in the class, respectively. For all events the number of complete observations increases with larger window size.

**Table 2:** The number of observations in the total dataset with all required features present within each of the different classes

Class	30 min	60 min	120 min
Stable	7,980 (32%)	11,183 (45%)	11,994 (51%)
SAE	835 (21%)	1,181 (29%)	1,315 (33%)
EWS6	1,965 (28%)	2,453 (35%)	2,617 (39%)
EWS8	312 (21%)	356 (25%)	376 (26%)
EWS10	54 (27%)	57 (28%)	63 (32%)

In the parentheses the percentage of observations with all required features present in relation to the total number of observations, i.e. including observations with any given number of features missing

**Fig. 4:**
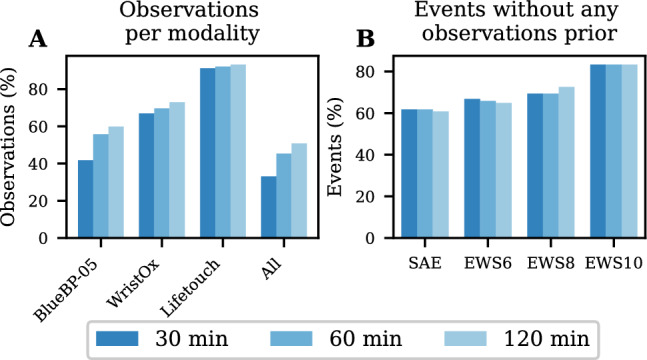
Available data in observations and events. **A**: Percentage of the observations containing data from each of the three devices used and all together. **B**: Percentage of events which have no complete observations, i.e. with all required features, in the period of 8 h prior to the event

For all window sizes, the percentage of complete observations in relation to the total number of observations is substantially higher for the stable class than any of the event classes. For all but two event/window size combinations, less than 1/3 of the observations computed are complete. The high amount of incomplete data is also supported by the two plots in Fig. 4.

Figure 4b shows that across all window lengths and events combinations, more than 60% of the events have no complete observations in the period of 8 h prior to the event. For the EWS10 events, this accounts for above 80% of the events. Figure 4a shows the same tendency of incomplete data in the full set of observations, where complete observations are only present in 33%, 45% and 51% for a window size of 30, 60, and 120 min, respectively. Of the three devices, the Lifetouch (HR and RR) has the highest on-time (91%, 92%, and 93%), the WristOx (SpO2) the second highest on-time (67%, 70%, 73%) and the BlueBP-05 (Sys. BP) the lowest (41%, 56%, 60%).

### Model performance on the evaluated cohorts

The classification results of the experiments conducted in each of the window sizes of 30 min, 60 min, and 120 min on the postoperative cohort, and 120 min for the AECOPD cohort are shown in Table 3. The results show good discriminative properties for the events based on EWS scores (AUROC: 0.772–0.993), with increasing AUROC following the magnitude of the score. For all EWS events the average TEW at FPR = 0.1 is at least 2.8 h, with the EWS10 events being at 5.5 h.

**Table 3:** Results of applying the proposed circadian KDE model to the evaluation set of postoperative patients

Window size	Event	Circadian setup		
		AUROC	TEW (FPR_0.1_)	Sen. (FPR_0.1_)
30 min	SAE	0.594 ± 0.014	2.5 ± 0.3 h	0.17 ± 0.06
	EWS6	0.772 ± 0.013	2.8 ± 0.2 h	0.40 ± 0.09
	EWS8	0.867 ± 0.010	3.4 ± 0.2 h	0.57 ± 0.10
	EWS10	0.993 ± 0.003	5.5 ± 0.0 h	1.00 ± 0.00
60 min	SAE	0.606 ± 0.015	2.5 ± 0.3 h	0.17 ± 0.06
	EWS6	0.776 ± 0.014	2.8 ± 0.2 h	0.41 ± 0.09
	EWS8	0.887 ± 0.008	3.4 ± 0.2 h	0.62 ± 0.07
	EWS10	0.992 ± 0.004	5.5 ± 0.0 h	1.00 ± 0.00
120 min	SAE	0.611 ± 0.019	2.6 ± 0.2 h	0.19 ± 0.05
	EWS6	0.796 ± 0.014	3.0 ± 0.2 h	0.45 ± 0.07
	EWS8	0.906 ± 0.009	3.8 ± 0.2 h	0.67 ± 0.06
	EWS10	0.991 ± 0.003	5.5 ± 0.0 h	0.98 ± 0.02
AECOPD 120 min	SAE	0.586 ± 0.011	1.5 ± 0.1 h	0.12 ± 0.02
	EWS6	0.734 ± 0.014	1.6 ± 0.1 h	0.29 ± 0.05
	EWS8	0.980 ± 0.002	2.4 ± 0.5 h	1.00 ± 0.01
	EWS10	–	–	–

Each value is listed the mean of 20 runs with random training and test set splits $\pm$ the 95% confidence interval of the mean. The TEW at FPR = 0.1denote the highest TEW value while the FPR is below 0.1

*h* hours, *sen* sensitivity

The discriminative ability between the stable observations and the SAEs was substantially lower (AUROC: 0.594–0.611), but still with a high average TEW at FPR = 0.1 of 2.5–2.6 h. The results show a small increase in performance with longer window size, which is seen for the metrics in all events apart from the EWS10 event. Therefore, the AECOPD cohort was evaluated with a window size of 120 min. From the results shown in Table 3 it can be seen that the results are similar to the postoperative cohort, although the EWS6 events were a bit lower and the EWS8 events a bit higher.

**Fig. 5:**
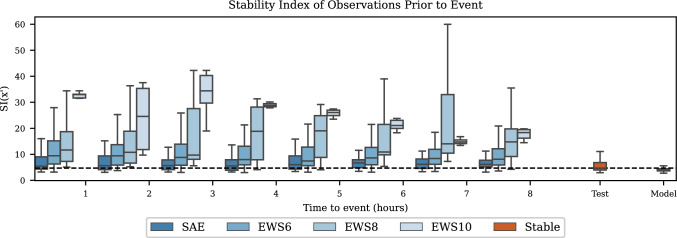
Illustration of the stability index prior to events and for stable test and training observations for the circadian model. The boxplots are ordered by the time to event from each observation, as indicated on the x-axis. The boxplot at ‘Test’ represents the stability index on the stable observations in the test set, and the boxplot at ‘Model’ represents the observations for which the model is created. The dashed line is at the median stability index of the stable observations in the test set. The plot is created from the 20 analyses conducted for the circadian model with a window size of 120 min. Whiskers represents the most extreme value within 1.5 times in inter quantile range and outliers outside this region are not shown in the plot

The boxplots in Fig. 5 show the stability index for each of the event classes in the 8 h prior to the events and the stable observations using a window size of 120 min. The plot shows a large difference in the distribution of the stable observations and the EWS events—especially EWS8 and EWS10 events—in the entire period. The boxplots for the SAEs show a higher resemblance with the stable observations without no substantial change throughout the 8 h.

**Fig. 6:**
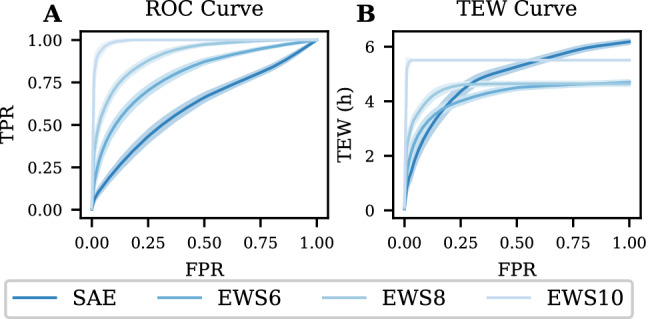
Classification results for the circadian model with window size of 120 min given as mean (solid line) and 95% confidence interval (shaded area). **A**: ROC curve for the four different event classes against the stable observations. **B**: TEW for the four event classes against the FPR for the stable observations. *ROC* Receiver Operator Characteristics, *TEW* Time of Early Warning, FPR False Positive Rate, TPR True Positive Rate

Figure 6 shows the ROC (Fig. 6A) and the TEW/FPR curve (Fig. 6B) for a window size of 120 min. The TEW/FPR curve show that the maximum value for the TEW given the dataset is 5.5 h achieved already at FPR < 0.1. The curves for the EWS6 and EWS8 events show a maximum of approximately 4 h and for the SAEs approximately 6 h.

### Applying the stability index on patient timelines

**Fig. 7:**
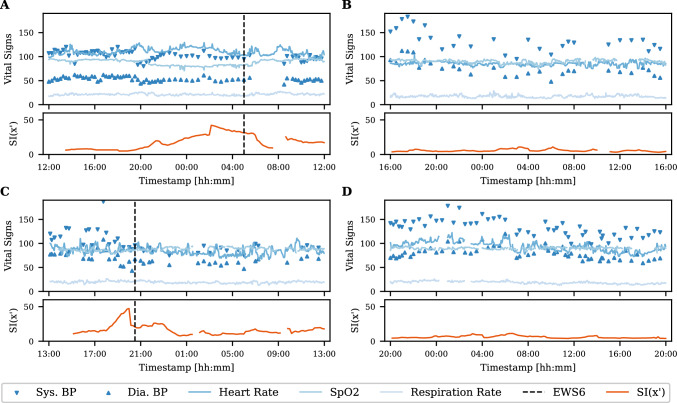
Visualization of the vital signs along with the stability index from the circadian KDE model in patients from the two cohorts. For each pair of plots, the upper plot shows the vital signs after moving median filtering, and the lower plot shows the stability index computed from observations with a window size of 120 min. **A**: Timeline for a postoperative patient prior and after an EWS10 event (marked by the dashed line). **B**: Timeline for a postoperative patient with stable physiology. **C**: Timeline for an AECOPD patient prior and after an EWS6 event (marked by the dashed line). **D**: Timeline for an AECOPD patient with stable physiology

We illustrated the application of the stability index model on a timeline of four patients’ vital signs from the postoperative and AECOPD cohort in Fig. 7. Each plot shows 24 h surrounding either an event (subfigure A and C) or a period from a patient in the stable class (subfigure B and D). For the presented patients experiencing an event, the SI is much higher than the patients without events. Whereas subfigures B and D, presenting stable patients, have continuing low stability indexes throughout, the patient with an EWS10 event (subfigure A) show increasing stability index lasting for approx. 8 h prior to the event. In subfigure C, representing an EWS6 event, it can be seen that the stability index also increases prior to the event, although this happens closer to the event.

## Discussion

In this study, we found that a simple KDE model using classic vital signs parameters could distinguish between observations originating from stable physiology and observations in the period prior to an event, though with different discriminative properties. The results further showed that the computed stability index allows for the identification of events hours prior to the event being noted by medical staff.

From the experiments conducted, the SAEs were the events that were the most difficult to distinguish from the stable observations. Compared to the EWS events (AUROC: 0.772–0.993), the SAEs yielded both lower AUROC (AUROC: 0.594–0.611) and TEW (TEW at FPR = 0.1: 2.5–2.6 h). Although the SAEs by definition represent a physiological deterioration, it is difficult to assume that the presence of SAEs will have any substantial effect on the vital signs given that they encompass both respiratory, circulatory, infectious or various other categories. Whereas other studies have previously considered events as clinical emergencies [12, 16], ICU admission [16, 24], or death [16, 24], this study includes much less severe events which fulfill the criteria for a SAE, but might not introduce a large physiological effect on the vital signs. In addition, events based on ICU admission or death are both dichotomic labels, whereas the SAEs used in this study are progressing by nature, which implies uncertainty in both the magnitude of the manifestation and the timestamp of the event.

Comparing the different window lengths, the results show a slight increase in AUROC with increasing window length (except for EWS10 events). This increase might be caused by multiple factors. Firstly, as the features are based on the mean value within the window, shorter periods of deviating vital signs, which expectedly can occur from physical activity during e.g. hygienic procedures, will have a larger impact on the features computed with a window length of 30 min than 60 and 120 min. This might lead to observations with a longer window length producing less false positives. Secondly the longer window lengths results in substantially more valid observations, which provides a larger basis for giving a classification. If the latter is true, the increase in performance with longer window lengths, might just be caused by the increased sample size and thus not indicate increased discriminative properties.

In the observations computed, BlueBP-05 and WristOx2 had most incomplete observations while the Lifetouch showed good up-time comparable with similar devices [33]. The results presented in Fig. 4 show, that in the period preceding an event, the proportion of incomplete observations (around 60%–80%) is higher than across all observations (around 51%), suggesting that, to a larger extent, devices are removed or unable to send information as patients deteriorate. Haahr-Raunkjær et al. reported discomfort as a major factor for missing data, when using wearable devices for measuring vital signs and, thus, missing observations are a major area of concern, as most medical events would go unnoticed based solely on the lack of data [15]. The TEW values are highly influenced by the data availability, as incomplete observations cannot be classified as events, which can lead to a decrease in the TEW not related to the predictive ability of the model. Therefore, if data were available in the entire period prior to the events, the TEW values would likely be higher than those presented here.

The ability to distinguish normal physiology from vital sign derangements leading up to an adverse clinical event could prove vital in the field of in-hospital vital sign monitoring. Late recognition of patient deterioration is associated with worse outcomes and with the current practice of manual measurements, it is proven that clinical deterioration can go unnoticed [34, 35]. Therefore, our proposed model could potentially provide medical staff with the opportunity to intervene earlier than what is possible with manual spot checks. We used the TEW at FPR = 0.1 as a measure for this to represent the earliest time that medical staff could be notified at the cost of a small false rate of alerting. For the EWS10 events, the results show medical staff could be notified, on average, 5.5 h in advance compared to the spot measurements. This is lower for the other events (around 2.5 h, 2.7 h and 3.3 for the SAEs, EWS6 and EWS8 events, respectively), but they still exhibit a considerable potential early warning. These findings correspond to the results of Colopy et al., who achieved slightly higher TEW, though on different events [12].

The largest limitation of the study is the many incomplete observations due to missing measurements from one or more of the devices. The proposed model was constructed in such a way that observations needed to be complete in order to compute a stability index. This led to the majority of the events not being preceded by any valid observations. In other studies, the procedure has been based on single parameters, thus limiting the issues of single devices [16, 17]. However, as we created the model with multiple parameters, we included any covariate information found between the parameters. We did not investigate the models’ discriminative properties on incomplete observations but designing the model to predict from a single device or at least fewer devices may improve in the number of events that can be identified and thus should be investigated.

In addition, the current study did not investigate an optimal threshold for classification or its implications. Any set threshold will lead to events going undetected and false alerts being produced. Therefore, the selection of a threshold criterion encompasses a larger analysis in which post-processing and other mitigations might be necessary, which is out of scope of this study.

With constantly increasing rate of which health care data are generated and the variety of this, health care is now transitioning into the big data era [10]. Continuous monitoring of patients’ vital signs will assist in this by producing previously unseen amounts of data from hospitalized patients, which further will allow for building complex models to identifying patterns of clinical importance. While complex analyses of data are worth exploring, there is a balance to be struck between giving accurate predictions, the volume of data available and maintaining a degree of interpretability. Simpler methods, like the one proposed in this paper, have merits if they can be easily explained to clinicians who are not well versed in big data methodology.

## Conclusion

This study found that it is possible to alert medical staff 5.5 h before a severe deviation in vital signs (EWS10 events) with an AUROC of 0.993, thus demonstrating the potential beneficial implications for patient safety and quality of treatment if proven implementable in a clinical setting. For the SAE events, the predictive capability was lower (AUROC of 0.611), with a potential TEW of 2.5 h. The stability index proposed demonstrates an easy-implementable way of continuously assessing the physiological stability of hospitalized patients, which allows the earlier detection of deterioration. In addition, the model showed increasing performance with increasing severity of the EWS events. The circadian KDE model showed fairly good generalization to a different patient cohort and, once the limitations have been addressed, the model could prove a valuable tool for medical staff.

## References

[1] Goldhill DR, White SA, Sumner A (1999). Physiological values and procedures in the 24 h before ICU admission from the ward: pre-ICU admission procedures. Anaesthesia.

[2] Berlot G, Pangher A, Petrucci L (2004). Anticipating events of in-hospital cardiac arrest. Eur J Emerg Med.

[3] McGloin H, Adam SK, Singer M (1999). Unexpected deaths and referrals to intensive care of patients on general wards. Are some cases potentially avoidable?. J R Coll Phys Lond.

[4] Smith GB, Prytherch DR, Schmidt P (2006). Hospital-wide physiological surveillance-a new approach to the early identification and management of the sick patient. Resuscitation.

[5] Royal College of Physicians (2017). National Early Warning Score (NEWS) 2: Standardising the assessment of acute-illness severity in the NHS.

[6] Bailey TC, Chen Y, Mao Y (2013). A trial of a real-time Alert for clinical deterioration in patients hospitalized on general medical wards. J Hosp Med.

[7] Pedersen NE, Rasmussen LS, Petersen JA (2018). A critical assessment of early warning score records in 168,000 patients. J Clin Monit Comput.

[8] Leenen JPL, Leerentveld C, van Dijk JD (2020). Current evidence for continuous vital signs monitoring by wearable wireless devices in hospitalized adults: systematic review. J Med Internet Res.

[9] Weenk M, Koeneman M, van de Belt TH (2019). Wireless and continuous monitoring of vital signs in patients at the general ward. Resuscitation.

[10] Webster CS, Scheeren TWL, Wan YI (2022). Patient monitoring, wearable devices, and the healthcare information ecosystem. Br J Anaesth.

[11] Clifton L, Clifton DA, Pimentel MAF (2014). Predictive monitoring of mobile patients by combining clinical observations with data from wearable sensors. IEEE J Biomed Health Inform.

[12] Colopy GW, Pimentel MAF, Roberts SJ, Clifton DA. Bayesian Gaussian processes for identifying the deteriorating patient. In: 2016 38th Annual International Conference of the IEEE Engineering in Medicine and Biology Society (EMBC). IEEE, Orlando, FL, USA. 2016; pp 5311–5314. 10.1109/EMBC.2016.7591926 28269459

[13] Pimentel MAF, Clifton DA, Clifton L (2013). Modelling physiological deterioration in post-operative patient vital-sign data. Med Biol Eng Comput.

[14] Elvekjær M, Rasmussen SS, Grønbæk KK (2022). Clinical impact of vital sign abnormalities in patients admitted with acute exacerbation of chronic obstructive pulmonary disease: an observational study using continuous wireless monitoring. Intern Emerg Med.

[15] Haahr-Raunkjær C, Mølgaard J, Elvekjaer M (2022). Continuous monitoring of vital sign abnormalities; association to clinical complications in 500 postoperative patients. Acta Anaesthesiol Scand.

[16] Eddahchouri Y, Peelen RV, Koeneman M (2022). Effect of continuous wireless vital sign monitoring on unplanned ICU admissions and rapid response team calls: a before-and-after study. Br J Anaesth.

[17] van Rossum MC, Vlaskamp LB, Posthuma LM (2022). Adaptive threshold-based alarm strategies for continuous vital signs monitoring. J Clin Monit Comput.

[18] Duus CL, Aasvang EK, Olsen RM (2018). Continuous vital sign monitoring after major abdominal surgery-Quantification of micro events. Acta Anaesthesiol Scand.

[19] Elvekjaer M, Aasvang EK, Olsen RM (2020). Physiological abnormalities in patients admitted with acute exacerbation of COPD: an observational study with continuous monitoring. J Clin Monit Comput.

[20] Mayer L, Rasmussen SS, Molgaard J et al. Prediction of Serious Adverse Events from Nighttime Vital Signs Values. In: 2022 44th Annual International Conference of the IEEE Engineering in Medicine & Biology Society (EMBC). IEEE, Glasgow, Scotland, United Kingdom. 2022; pp 2631–2634. 10.1109/EMBC48229.2022.9871778 36086507

[21] Kristinsson ÆÖ, Gu Y, Rasmussen SS (2022). Prediction of serious outcomes based on continuous vital sign monitoring of high-risk patients. Comput Biol Med.

[22] Colopy GW, Pimentel MAF, Roberts SJ, Clifton DA. Bayesian optimisation of Gaussian processes for identifying the deteriorating patient. In: 2017 IEEE EMBS International Conference on Biomedical & Health Informatics (BHI). IEEE, Orland, FL, USA. 2017; pp 85–88.

[23] Izquierdo LM, Nino LF, Prieto Rojas J. Modeling the vital sign space to detect the deterioration of patients in a pediatric intensive care unit. In: Brieva J, Lepore N, Romero Castro E, Linguraru MG (eds) 16th International Symposium on Medical Information Processing and Analysis. SPIE, Lima, Peru. 2020; p 31.

[24] Pimentel MAF, Clifton DA, Clifton L, et al. Vital-Sign Data Fusion Models for Post-Operative Patients. In: Proceedings of the International Conference on Bio-inspired Systems and Signal Processing. SciTePress - Science and and Technology Publications, Vilamoura, Algarve, Portugal. 2012; pp 410–413.

[25] van Goor HMR, van Loon K, Breteler MJM (2022). Circadian patterns of heart rate, respiratory rate and skin temperature in hospitalized COVID-19 patients. PLoS ONE.

[26] Davidson S, Villarroel M, Harford M (2020). Vital-sign circadian rhythms in patients prior to discharge from an ICU: a retrospective observational analysis of routinely recorded physiological data. Crit Care.

[27] Sow D, Biem A, Jimeng Sun, et al. Real-time prognosis of ICU physiological data streams. In: 2010 Annual International Conference of the IEEE Engineering in Medicine and Biology. IEEE, Buenos Aires. 2010; pp 6785–6788. 10.1109/IEMBS.2010.5625983 21095840

[28] European Medicines Agency (EMA). Guideline for good clinical practice E6(R2). 2018.

[29] Region Hovedstaden Early Warning Score (EWS) - systematisk observation og risikovurdering af indlagte patienter samt dertil hørende handlingsalgoritme. https://vip.regionh.dk/VIP/Admin/GUI.nsf/Desktop.html?open&openlink=http://vip.regionh.dk/VIP/Slutbruger/Portal.nsf/Main.html?open&unid=X87330D22C49DFAA8C12579D000464B81&dbpath=/VIP/Redaktoer/RH.nsf/&windowwidth=1100&windowheight=600&windowtitle=S%F8g. Accessed 11 Nov 2022.

[30] Härdle W, Werwatz A, Müller M, Sperlich S (2004). Nonparametric and semiparametric models.

[31] Scott DW (1992). Multivariate density estimation: theory, practice, and visualization.

[32] Silverman BW (1998). Density estimation for statistics and data analysis.

[33] Breteler MJM, KleinJan EJ, Dohmen DAJ (2020). Vital signs monitoring with wearable sensors in high-risk surgical patients. Anesthesiology.

[34] Thomson R (2008). Safer care for the acutely ill patient: learning from serious incidents.

[35] National Patient Safety Agency (2007). Recognising and responding appropriately to early signs of deterioration in hospitalised patients: November 2007.

